# Discrepancies Between Automated and Manually Corrected RNFL Measurements in Papilledema Associated with Idiopathic Intracranial Hypertension

**DOI:** 10.51894/001c.164047

**Published:** 2026-07-31

**Authors:** Vineet Kumar, Amaka Okeke, Luke Barrick, John Reaves, Grant Goodfellow, Laiba Khalil, David Kaufman

**Affiliations:** 1 College of Osteopathic Medicine, Michigan State University

## 26

### Introduction

Automated peripapillary retinal nerve fiber layer (RNFL) segmentation on optical coherence tomography (OCT) is widely used for monitoring optic neuropathies. However, in papilledema associated with Idiopathic Intracranial Hypertension (IIH), segmentation algorithms frequently fail, potentially producing inaccurate RNFL measurements that may misguide clinical decision-making. Although segmentation errors in papilledema have been described, the degree of deviation from systemic manual correction across varying edema severities has not been formally quantified. We aimed to perform a preliminary evaluation of agreement between automated and manually corrected RNFL measurements in IIH and to characterize the magnitude and distribution of segmentation discrepancies across varying stages of papilledema severity.

### Hypothesis

We hypothesized that automated RNFL measurements would show greater deviation with increasing papilledema severity.

### Methods

We conducted a retrospective observational study of 31 patients comprising 32 IIH eyes and 30 control eyes evaluated at a tertiary neuro-ophthalmology clinic. Inclusion criteria for IIH eyes included age 18–50 years, confirmed IIH diagnosis, and high-quality spectral-domain OCT scans (signal strength ≥20; ART ≥100). Eyes with other optic neuropathies or ungradable scans were excluded. Among the 32 IIH eyes, papilledema severity was graded by a masked neuro-ophthalmologist using the Frisén scale. Six eyes were included in each grade category (I–V), with one additional eye classified as Grade I. Global peripapillary RNFL thickness values were extracted from automated segmentation outputs for controls and each grade. Automated segmentation boundaries were reviewed and manually corrected on the Heidelberg Spectralis platform by a masked clinical fellow.

### Results

Sixty-two eyes were analyzed. Discrepancy between automated and corrected RNFL measurements increased with papilledema severity. Deviations between automated and manual measurements were small in lower grades (Grade 1: 2.6 µm; Grade 2: 18.5 µm; Grade 3: 27.2 µm) but markedly larger in severe papilledema, with Frisén grades 4–5 requiring corrections of 194 µm compared with controls (p < 0.001).

### Discussion

Inaccuracies in papilledema measurement may be underrecognized, and limitations in automated OCT segmentation algorithms could influence treatment strategies in IIH suboptimally. RNFL measurement errors may misrepresent disease severity or progression, emphasizing the need for manual review of OCT segmentation.

